# Eradication of intractable malignant ascites by abdominocentesis, reinfusion of concentrated ascites, and adoptive immunotherapy with dendritic cells and activated killer cells in a patient with recurrent lung cancer: a case report

**DOI:** 10.1186/1752-1947-2-372

**Published:** 2008-12-04

**Authors:** Hideki Kimura, Toshihiko Iizasa, Aki Ishikawa, Mitsuru Yoshino, Masato Shingyouji, Masaki Kimura, Tetushi Hirata, Akiko Odaka, Keiko Matsubayasi

**Affiliations:** 1 Department of Thoracic Diseases, Chiba Cancer Center, Chiba, Japan; 2 Division of Biochemistry, Chiba Cancer Center Research Institute, Chiba, Japan; 3 Department of Clinical Pathology, Chiba Cancer Center, Chiba, Japan; 4 Department of Hematology, Chiba Cancer Center, Chiba, Japan

## Abstract

**Introduction:**

Malignant ascites is often a sign of a terminal stage in several malignant diseases. To control ascites, drainage and intra-abdominal chemotherapy are often used in those patients but eradication of ascites is difficult and prognosis is poor.

**Case presentation:**

A 55-year-old woman was admitted to our hospital on 26 January 2007 with dyspnea, abdominal distention and oliguria. Abdominocentesis revealed peritoneal carcinomatosis resulting from abdominal recurrence from lung cancer. To alleviate the dyspnea and abdominal distention, we drained the ascites aseptically and infused them intravenously back into the patient after removal of tumor cells by centrifugation, and then concentration by apheresis. After the drainage of ascites, we intraperitoneally infused activated killer cells and dendritic cells from the patient's tumor-draining lymph nodes, together with 4.5 × 10^5^U interleukin-2 in 50 ml saline by 2.1 ml/hour infuser balloon.

Drastic decreases in the tumor cell count and in ascite retention were observed after several courses of ascites drainage, intravenous infusion and intraperitoneal immunotherapy. The plasma protein level was maintained during the treatment notwithstanding the repeated drainage of ascites. Cell surface marker analysis, cytotoxic activities against autologous tumor cells and interferon-gamma examination of ascites suggested the possibility that these effects were mediated by immunological responses of activated killer cells and dendritic cells infused intraperitoneally.

**Conclusion:**

Combination of local administration of immune cells and infusion of concentrated cell free ascites may be applicable for patients afflicted with refractory ascites.

## Introduction

Malignant ascites is often a sign of terminal stage in several malignant diseases. It represents one of the most common causes of death in patients with digestive malignancies with an overall median survival of 3 to 6 months [1-6]. These patients are considered as being in the terminal phase of their diseases and they receive palliative or symptomatic treatments. Abdominocentesis and drainage alleviate symptoms caused by ascites such as abdominal distention, dyspnea and oliguria, but cause hypovolemia, hypoproteinemia and general malaise. To control ascites, drainage and intra-abdominal chemotherapy are often employed for those patients, but eradication of ascites is difficult and the prognosis is poor.

In this study, we report on a patient with peritoneal carcinomatosis caused by a recurrence of lung cancer that was successfully treated with abdominocentesis, reinfusion of concentrated ascites and adoptive immunotherapy with dendritic cells and activated killer cells.

## Case presentation

A 52-year-old woman underwent right upper lobectomy of primary lung adenocarcinoma after induction chemotherapy on 2 November 2004. Histological examination revealed pleural dissemination, intrapulmonary metastasis (PM2) and mediastinal lymph node metastasis (pT4N2M1 stage IV). She received nine courses of adjuvant chemotherapy, 7 of intrathoracic immunotherapy (1.82 × 10^10 ^cells) and 12 of intravenous immunotherapy (5.6 × 10^10 ^cells) with dendritic cells and activated killer cells (DC+AK) obtained from long-term tissue cultures of regional lymph nodes of lung cancer. She was admitted to our hospital for dyspnea, abdominal distention and oliguria on 26 January 2007. The abdominocentesis revealed peritoneal carcinomatosis resulting from abdominal recurrence from lung cancer.

To alleviate dyspnea and abdominal distention, we drained ascites aseptically and infused it back to the patient intravenously after removal of tumor cells by centrifugation and concentration by apheresis (apheresis monitor KM-8900, Kuraray Medical Co. Tokyo Japan). Before infusion, the endotoxin of ascites was quantified and those samples with less than 3 pg/ml endotoxin were used.

The methods for *in vitro *culture of dendritic cells and activated killer cells have been described elsewhere [7]. Briefly, regional lymph nodes with no metastasis obtained during surgery for the primary lung cancer were minced aseptically into blocks of about 1 mm^3 ^and suspended in KBM-400 serum-free lymphocyte culture medium (Kojin Bio, Tokyo, Japan) with 400 IU interleukin 2 (IL2; Proleukin Chiron B.V., Amsterdam, Netherlands). Two weeks later, the tumor draining lymph node (TDL) tissue culture was transferred to a CO_2 _gas permeable culture bag, and every 3 to 4 days, fresh medium was added. When dendritic cells and activated killer cells were released from the lymph node tissue, cells were harvested and tissue culture continued until the release of new cells ceased (dormant phase). The tissue started to produce DC+AK cells when one to two billion (1–2 × 10^9^) peripheral blood lymphocytes were added to the culture at this dormant phase. Using this procedure, we obtained a total of 1.54 × 10^11 ^DC+AK cells.

We also used cells from ascites (tumor infiltrating lymphocytes, TIL) obtained after DC+AK immunotherapy. The TIL were washed three times in PBS and cultured in KBM-400 lymphocyte medium for 2 weeks.

As a control for cytotoxic tests, we cultured regional lymph nodes from another patient with lung cancer (48-year-old man, T4N2M0, stage IIIB adenocarcinoma) in IL2 for 1 month. The significant cytotoxic activity of the activated killer cells of this patient (MT-116) had already been confirmed against autologous tumor cells.

Cytological examination of the ascites before and after treatment was carried out using Papanicolaou stain. We also examined quantitatively the numbers of tumor cells in ascites before and after the treatment.

Cytokine concentration of IFN-gamma, TGF-beta, and IL12 in ascites was measured by a Sandwich ELISA Kit (Chemicon International, Inc., CA, USA) before, and 8 hours, 24 hours, 48 hours and 5 days after the first intraperitoneal immunotherapy.

The cytotoxic activity of TIL obtained from ascites was determined by the ^51^Cr-releasing test. One million tumor cells were labeled with 3.7MBq sodium chromate-^51^Cr (Na_2_^51^CrO_4_; Daiichi Radioisotope Lab. Ltd., Tokyo, Japan) for 45 minutes, washed three times with culture medium and suspended at 1 × 10^5^/ml. Effector lymphocytes obtained from ascites after immunotherapy were cultured in IL2 for 2 weeks, washed and counted. The cells were suspended at 1 × 10^7 ^cells/ml. In sterile glass tubes, 0.2 ml samples of labeled tumor cells (2 × 10^4^) were mixed with serial dilutions of effector cells (2 × 10^6 ^to 2.5 × 10^5^) in 0.2 ml of medium. The effector target cell mixture was incubated at 37°C in a 5% CO_2 _atmosphere for 4 hours. Two milliliters of cold medium was added at the termination of incubation, centrifuged at 900 g for 10 minutes and the radioactivity of the supernatant was examined with a gamma counter (Auto-well Gamma System; ARC-370, Aloka, Tokyo, Japan). The cytotoxic activity was calculated with the following formula:

Cytotoxic activity (%) = (CPM of test – CPM of medium control)/(CPM of Maximum control – CPM of medium control) ×100,

where medium control was measured in medium alone without effector cells and maximum control was counted in 0.2 ml of 1 N HCl instead of effector cells. All of the tests were performed in quadruplicate.

The cell surface markers of mononuclear cells were determined by a fluorescence-activated cell sorter with various monoclonal antibodies: CD3, CD4, CD8, CD83, and HLA-DR.

**Figure 1:**
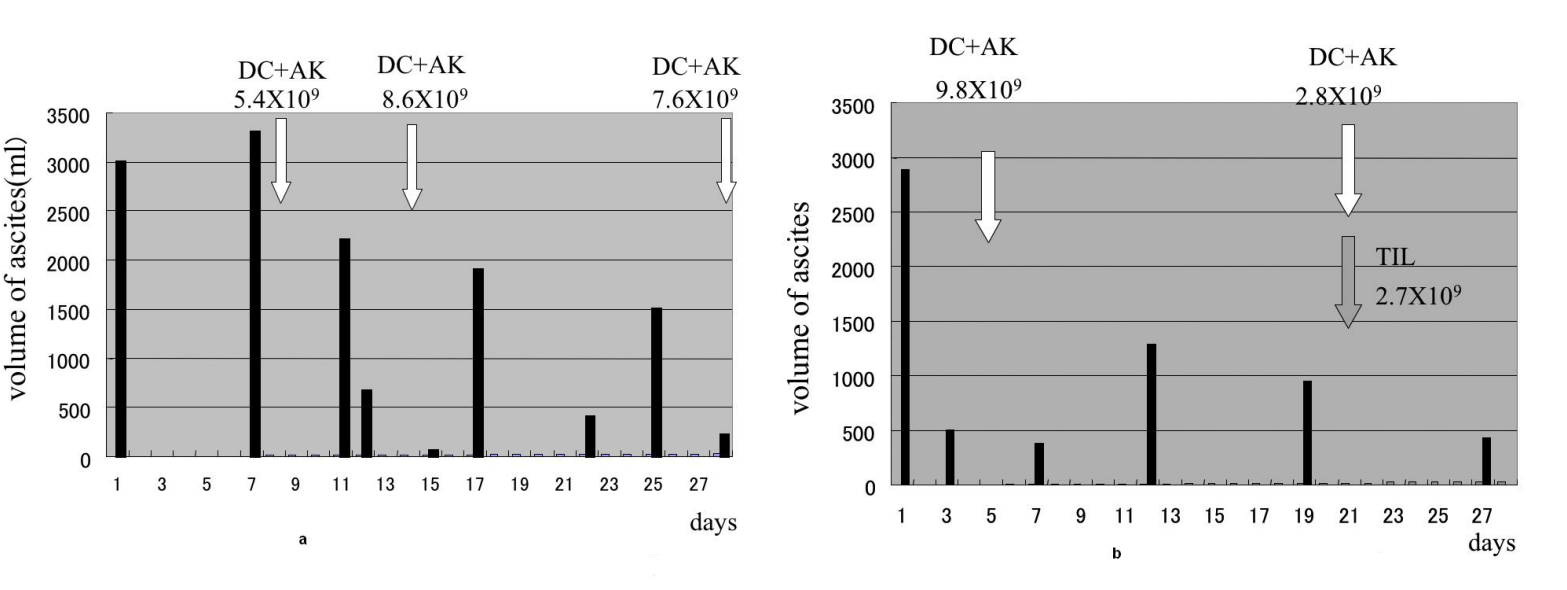
**Volume of ascites and number of transferred cells**: (a) Volume of ascites drained and number of DC+AK cells transferred into the hypogastric area. From 16 February 2007 (day 1), we started to drain 3000 ml of ascites and transferred 5.4 × 10^9 ^DC+AK cells on day 8. Drainage and intraperitoneal immunotherapy in the hypogastric area continued for 1 month. A total of 13,222 ml of ascites was aspirated. Black bars indicate the volume of ascites, and white arrows, the date of DC+AK immunotherapy. (b) Volume of ascites drained and number of DC+AK cells transferred into the epigastric area. From 23 March 2007 (day 1), we started to drain 2800 ml of ascites and transferred 9.8 × 10^9 ^DC+AK cells on day 5. Drainage and intraperitoneal immunotherapy in the epigastric area continued for 1 month. A total of 6000 ml of ascites was aspirated. Black bars indicate the volume of ascites, and white arrows, the date of DC+AK immunotherapy. The gray arrow indicates the date of TIL immunotherapy.

From 16 February 2007, we drained 3000 ml of ascites from the hypogastric area every week for 1 month. A total of 13,222 ml of ascites was aspirated from this area (Figure 1a). Then, from 23 March 2007, we drained a total of 6000 ml of ascites from the epigastric area over a period of 3 weeks (Figure 1b).

A total of 2480 ml of concentrated ascites was infused back into the patient six times (Figures 1a and 1b) and this was equivalent to 4808 ml of 4.4% albumin. The constituents of ascites before and after apheresis are listed in Table 1. The protein and albumin of ascites were concentrated 3 to 4 times but electrolytes, blood urea nitrogen (BUN), creatinine and uric acid were dialyzed through the membrane. The plasma protein concentration was maintained between 5.5 and 6.2 g/dl during the patient's hospital stay notwithstanding the repeated drainage of ascites (Table 2). Chills and fever for 20 to 30 minutes beginning soon after the initiation of infusion were the only side effects of this treatment.

**Table 1:** Constituents of ascites before and after apheresis

	23 February		13 March	
	
	Before	After	Before	After
Total protein (g/dl)	4.71	21.19	4.73	14.04
Albumin (g/dl)	2.78	12.92	2.59	7.97
BUN (mg/dl)	18.7	12.6	12.7	3.7
Creatinine (mg/dl)	0.65	0.47	0.57	0.2
Uric acid (mg/dl)	7.6	6.7	6.9	2.9
Total cholesterol (mg/dl)	135	421	125	370
AST (IU/l)	17	79	17	74
ALT (IU/l)	6	25	6	14
LDH (IU/l)	559	2336	517	2124
Gamma-GTP (IU/l)	48	61	39	48
ALP (IU/l)	787	825	464	589
Na (mEq/l)	138	141	140	146
K (mEq/l)	3.5	2.7	3.1	1.2
Cl (mEq/l)	102	85	102	105
Total Bil (mg/dl)	0.5	2.6	1.5	4.7
Direct Bil (mg/dl)	0.1	0.3	0.4	0.6
CRP (mg/dl)	1.9	8	1.4	5.4
Endotoxin (pg/ml)	2.23	<3	1.87	<3

**Table 2:** Plasma protein and albumin levels during treatment

	19 February	26 February	9 March	12 April
Total protein (g/dl)	6.2	6	6	6.3
Albumin (g/dl)	3.3	3.4	3.3	3.3

Surface markers of cells obtained from long-term tissue culture of tumor-draining lymph nodes (TDL) of the patient and tumor-infiltrated lymphocytes (TIL) obtained from the ascites after immunotherapy, were determined by fluorescence-activated cell-sorting (FACS) using monoclonal antibodies (Table 3). Cells from TDL consist mainly of CD8-positive and CD4-positive T cells and CD83-positive dendritic cells. TIL consists mainly of CD8-positive T cells.

**Figure 2:**
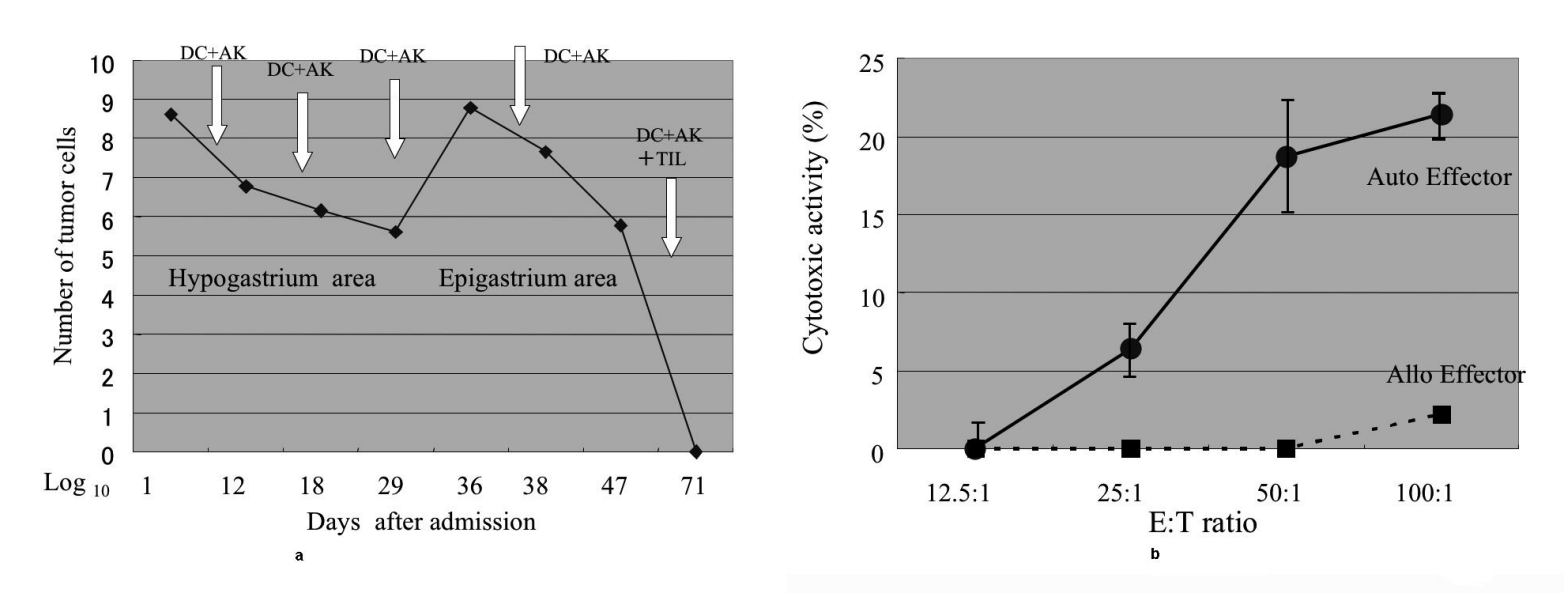
(a) Number of tumor cells obtained from ascites. From 16 February 2007 (day 1), we started to drain 3000 ml of ascites and counted the number of tumor cells in the hypogastric area. After the second drainage of ascites, we infused DC+AK cells intraperitoneally with IL2. Three days later, 2200 ml of ascites was drained and the number of tumor cells was counted. A drastic decrease in the tumor cell count (1/77) was observed: from 4.6 × 10^8 ^cells to 6 × 10^6 ^cells. From 23 March (day 36), we initiated drainage from the epigastric area. The number of tumor cells decreased from 6.6 × 10^8 ^(day 36) to 0 (day 71) after the fifth immunotherapy with DC+AK cells and TIL. (b) Cytotoxic activity of autologous and allogeneic effector cells. Cytotoxic activity of TIL was examined by the ^51^Cr-releasing test. Tumor cells obtained from ascites were labeled with Na_2_^51^CrO_4_, incubated with autologous effector (TIL) cells and allogeneic effector (MT-116) cells. ^51^Cr release was counted with a gamma counter. TIL showed a high cytotoxic activity against autologous tumor cells (black filled diamond), but MT-116 did not (black filled square).

After the second drainage of ascites, we infused 5.4 × 10^9 ^DC+AK cells intraperitoneally accompanied by 4.5 × 10^5^U IL2 in a 50 ml saline infuser balloon (2.1 ml/hour; Surefuser A; Nipro, Tokyo, Japan, Figure 1). Three days later, 2200 ml ascites was drained and the number of tumor cells was counted. A drastic decrease in the tumor cell count (1/77) was observed; from 4.6 × 10^8 ^cells in 3000 ml to 6 × 10^6 ^cells in 2200 ml ascites (Figure 2a). The third treatment with 7.6 × 10^9 ^DC+AK and IL2 revealed complete disappearance of the ascitic fluid in the hypogastric area. The treatment of the epigastric area with 9.8 × 10^9 ^DC+AK and 4.5 × 10^5^U IL2 reduced the tumor cell count from 6.6 × 10^8 ^to 6 × 10^5 ^(1/1100; Figures 1b and 2a). After the first treatment of the epigastric area, we cultured lymphocytes obtained from ascites in KBM-400 serum-free lymphocyte culture medium for 2 weeks. Cytological examination, analysis of the cell surface markers (Table 3) and a cytotoxicity test against autologous tumor cells (Figure 2b) indicated that TIL consists of cytotoxic T cells without tumor cell contamination. We inoculated 2.8 × 10^9 ^TIL along with 2.7 × 10^9 ^DC+AK intraperitoneally (Figure 1b). After this treatment, ascite retention stopped and tumor cells were not found cytologically.

**Table 3:** Cell surface markers of DC+AK and TIL

	DC+AK	TIL
CD3	99.2	98.9
CD8	38.2	97.3
CD4	68.2	7
HLA-DR	97.9	95.7
CD83	19.1	2

The cytokine concentration of IFN-gamma (Table 4), TGF-beta, and IL12 in ascites were measured before, and 8 hours, 24 hours, 48 hours and 5 days after the first intraperitoneal immunotherapy. Although the concentrations of TGF-beta and IL12 did not change during the period (data not shown), that of IFN-gamma increased significantly up to 400 pg/ml 8 hours after the initiation of the first immunotherapy. The concentration of IFN-gamma decreased gradually with time to 130 pg/ml 5 days later.

**Table 4:** Concentration of IFN γ in the ascites (pg/ml ± SD*)

Before	8 hours	24 hours	48 hours	5 days
23 ± 4	401 ± 27	383 ± 1	272 ± 9	130 ± 14

*SD, standard deviation.

The cytotoxic activity of TIL and other effector cells (MT-116) against tumor cells obtained from ascites was determined (Figure 2b). TIL showed a high cytotoxic activity against autologous target cells, but allogeneic effector cells (MT-116) failed to show activity against the same target cells.

**Figure 3:**
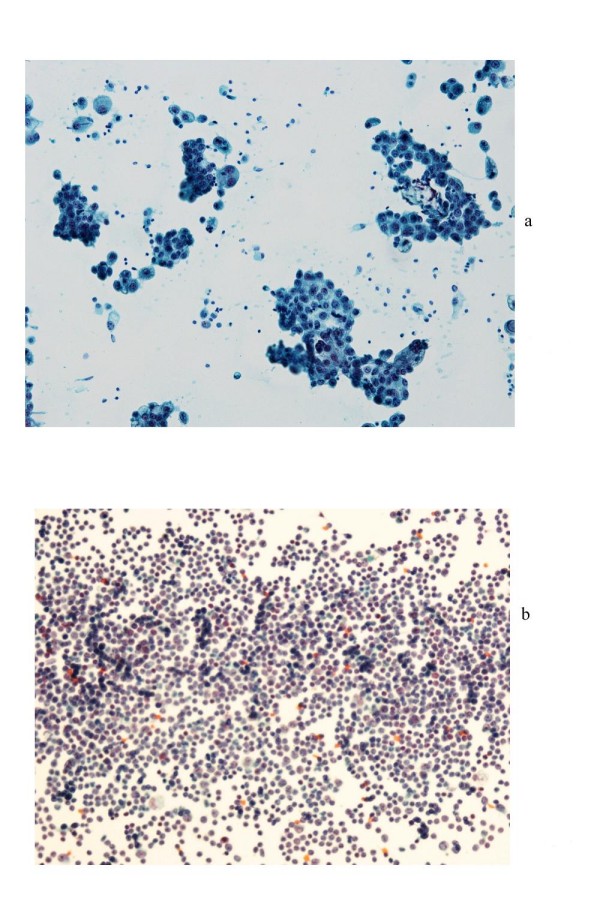
**Cytological findings of ascites**. (a) Papanicolaou stain of ascites on 9 February 2007 (before treatment). Carcinoma cells varying in size with prominent nucleoli form papillary pattern clusters. (b) Papanicolaou stain of ascites on 18 April 2007 (after treatment). Most of the infiltrates are small lymphocytes and carcinoma cells are not present. Cell surface marker analysis indicated that most of the cells were CD4- and CD3-positive T cells (data not shown).

Cytological examination was carried out before and after intraperitoneal immunotherapy. Before the treatment, ascites consisted exclusively of carcinoma cells varying in size with prominent nucleoli forming papillary pattern clusters (Figure 3a). After treatment, most of the cells turned out to be lymphocytes. Cell surface marker analysis indicated that most of the infiltrates were CD4-, and CD3-positive T cells (Figure 3b). The patient survived for more than 10 months after the initiation of therapy.

## Discussion

In this report, we present a case in which intractable malignant ascites was successfully treated by dendritic cell-activated killer cell immunotherapy and infusion of cell-free concentrated ascites to the patient. Malignant ascites that causes dyspnea, abdominal distention and retention of water in the body is often a sign of the terminal stage of cancer. Drainage of ascites induces a transient relief of symptoms but also induces hypoproteinemia, hypovolemia and a further retention of ascites. To maintain the serum protein level and give longer relief from the symptoms, we infused concentrated cell-free, endotoxin-free ascites back into the patient. By this procedure, the serum protein level was maintained between 5.5 and 6.2 g/dl, notwithstanding the repeated drainage of ascites once a week for 2 months. Chills and fever for 20 to 30 minutes beginning soon after the initiation of infusion, were the only side effects of this treatment. There were no changes of serum BUN, creatinine, electrolyte, bilirubin, or transaminase after the infusion of concentrated ascites. Since tumor cells were removed by centrifugation and filtration through a PS filter, pulmonary metastasis was not observed after the treatment.

This patient received 9 courses of adjuvant chemotherapy, 7 courses of intrathoracic immunotherapy (1.82 × 10^10 ^DC+AK cells) and 12 courses of intravenous immunotherapy (5.6 × 10^10 ^DC+AK cells) obtained from long-term tissue cultures of the regional lymph nodes of lung cancer. Despite the pleural dissemination, intrapulmonary metastasis (PM2) and mediastinal lymph node metastasis (T4N2M1 stage IV), intrathoracic recurrence was prevented for 2 years. Peritoneal recurrence may result from an insufficient immunotherapeutic effect in the abdominal cavity. Intravenous and intrathoracic inoculation of immune cells causes accumulation of effector cells in the thoracic cavity, but not in the abdominal cavity. Intra-abdominal inoculation of dendritic cells and activated killer cells decreased the number of tumor cells drastically. The precise mechanisms of eradication of tumor cells by the adoptive transfer of activated killer T cells and DCs are not clear, but it may be mediated mainly by the immune responses of cytotoxic killer T cells since an abrupt increase in IFN-gamma in ascites occurred after the treatment, cytotoxic activity of infiltrating T cells was detected against autologous tumor cells in ascites, and most of the cells recovered from the ascites after treatment were CD3-, CD4-, and CD8-positive T cells.

Although it is very difficult to apply these treatments to other patients with lung cancer, because recurrence only in the abdominal cavity as malignant ascites is very rare, further study is necessary to analyze the efficacy and mechanisms of this exploratory treatment.

## Conclusion

To control ascites, drainage and intra-abdominal chemotherapy are applied in most cases. However, complete eradication of ascites is difficult, and the prognosis of advanced cases of ascites is generally 2 to 3 months. The combination of local administration of immune cells and infusion of concentrated cell-free ascites may be applicable for patients afflicted with refractory ascites.

## Abbreviations

DC+AK: dendritic cells and activated killer cells; ELISA: enzyme-linked immunosorbent assay; IFN-gamma: interferon gamma; IL2: interleukin 2; IL12: interleukin 12; TDL: tumor draining lymph nodes; TGF-beta: transforming growth factor beta; TIL: tumor infiltrating lymphocytes.

## Consent

Informed consent for this therapy was obtained from the patient. She died 10 months after the initiation of this therapy. We obtained written informed consent for publication of this case report from the patient's next-of-kin. A copy of the written consent is available for review by the Editor-in-Chief of this journal.

## Competing interests

The authors declare that they have no competing interests.

## Authors' contributions

The authors contributed equally to the treatment and presentation.
